# Intimate partner violence and Musculoskeletal injury: bridging the knowledge gap in Orthopaedic fracture clinics

**DOI:** 10.1186/1471-2474-14-23

**Published:** 2013-01-15

**Authors:** Sheila Sprague, Kim Madden, Sonia Dosanjh, Katelyn Godin, J Carel Goslings, Emil H Schemitsch, Mohit Bhandari

**Affiliations:** 1 Department of Clinical Epidemiology and Biostatistics, McMaster University, 293 Wellington St. N Suite 110, L8L 8E7, Hamilton, ON, Canada; 2 Global Research Solutions, 3228 South Service Road, Suite 206, L7N 3H8, Burlington, ON, Canada; 3 Trauma Unit, Department of Surgery, Academic Medical Center, University of Amsterdam, Meibergdreef 9, 1105 AZ, Amsterdam, The Netherlands; 4 Division of Orthopaedic Surgery, St. Michael’s Hospital, 30 Bond Street, M5B 1W8, Toronto, ON, Canada; 5 Division of Orthopaedic Surgery, Department of Surgery, McMaster University, 293 Wellington St. N Suite 110, L8L 8E7, Hamilton, ON, Canada

**Keywords:** Intimate partner violence, Domestic violence, Identification program, Orthopaedic surgery, Musculoskeletal injury

## Abstract

Intimate partner violence (IPV) is a serious health issue. There have been widespread research efforts in the area of IPV over the past several decades, primarily focusing on obstetrics, emergency medicine, and primary care settings. Until recently there has been a paucity of research focusing on IPV in surgery, and thus a resultant knowledge gap. Renewed interest in the underlying risk of IPV among women with musculoskeletal injuries has fueled several important studies to determine the nature and scope of this issue in orthopaedic surgery. Our review summarizes the evidence from surgical research in the field of IPV and provides recommendations for developing and evaluating an IPV identification and support program and opportunities for future research.

## Background

Intimate partner violence (IPV) is a serious global health issue and is a large source of preventable morbidity and mortality among women [1]. It is characterized by a pattern of assaultive and coercive behaviours, including physical, sexual, and psychological attacks as well as economic coercion committed by both men and women against their partners [2]. Consequences of IPV include a host of physical and mental health problems documented in numerous studies [3]. The economic costs of IPV against women are substantial. Max and colleagues reported that IPV against women costs $5.8 billion, considering expenditures for increased medical care, mental health services, and lost productivity stemming from injury and premature death [4]. These large health consequences and costs to the health care system have led to widespread research efforts in the area of IPV over the past several decades, primarily focusing on obstetrics, emergency medicine, and primary care settings [5].

Until recently, there has been a paucity of research focusing on IPV in the realm of orthopaedic surgery. Renewed interest in the underlying risk of IPV among women with musculoskeletal injuries has fueled several important studies [6-13] to determine the nature and scope of this issue in orthopaedic surgery. Because of the high prevalence of IPV in orthopaedics [11], the evidence that patients rarely have their IPV documented by emergency department staff [14], and because orthopaedic surgeons see patients several times for follow up appointments, often when they are not in acute pain (as opposed to the emergency department), orthopaedic surgeons are in an ideal position to help victims of IPV [15]. This information has led several organizations to recommend that orthopaedic surgeons take a lead role in IPV identification and offering assistance to victims of IPV including the Canadian Orthopaedic Association and the American Academy of Orthopaedic Surgeons [15-17].

We present an overview of the current knowledge in the field of IPV and orthopaedics as well as where the knowledge gaps lie and we propose a method of developing a support program for IPV victims in orthopaedic fracture clinics based on the best available evidence.

## Review

### What do we know about intimate partner violence and musculoskeletal injury?

The field of orthopaedics has historically been closely involved in the identification and management of cases of child abuse involving physical injuries. Orthopaedic research in the area of child abuse and injuries began as early as 1974 with a description of battered child syndrome [18]. However, descriptions of IPV and musculoskeletal injuries did not appear in the orthopaedic literature until 1993 [19]. Varvaro and Lasko described the most common physical injuries from IPV as contusions, abrasions/lacerations, and fractures/strains/sprains with most injuries occurring on the face, neck, head, extremities, or in multiple locations [19]. Bhandari and colleagues evaluated 263 women referred to a domestic violence therapy and advocacy center and found that the most prevalent forms of abuse were emotional (84%), psychological (68%), physical (43%), sexual (41%), and financial (38%) [6]. Among those women who reported physical abuse, 46% sought medical attention. The authors identified 144 injuries in the 218 women who experienced physical abuse. Head and neck injuries were the most common, followed by musculoskeletal injuries, which included sprains, fractures and dislocations, and foot injuries [6]. The authors concluded that recognizing musculoskeletal injuries in women as a potential result of IPV is warranted [6].

A recent systematic review and meta-analysis examined the pattern of physical injury associated with IPV in women presenting to emergency rooms [7]. The association between head, neck, and facial injuries and IPV was higher among studies that excluded women with verifiable injuries such as witnessed falls or motor vehicle accidents. Thoracic, abdominal, or pelvic injuries were generally non-specific for IPV whereas upper extremities were suggestive of non-IPV etiology [7]. The authors cautioned that the quality of evidence pertaining to thoracic, abdominal, and extremity injuries may be limited by methodological issues and lack of data to a greater extent than findings that pertained to head, neck, and facial injuries [7].

A recent meta-analysis examined the prevalence of IPV across different medical specialities [20]. The authors included 37 articles in their study and found that most studies took place in family medicine clinics (15/37, 40.5%) and emergency departments (12/37, 32.4%). Pooled prevalence was reported for emergency (lifetime: 38%; one year: 19.9%) and family medicine (lifetime: 40%, one year: 19.5%). Data from other specialty areas suggested that from 43% (obstetrics and gynecology) to 73% (addiction recovery) of women experienced IPV during their lifetime of which 4% (pediatric emergency) to 21% (obstetrics and gynecology) of women experience physical abuse. This review did not find any data examining the prevalence of IPV in orthopaedic fracture clinics and identified this as a gap in the literature.

A cross-sectional study of women reporting to two trauma centres in Ontario found that one third of the respondents had experienced IPV in the last twelve months [11]. Emotional abuse was the most prevalent form of abuse (30.5%), followed by physical abuse (8.5%), and sexual abuse (3.3%) [11]. Seven women (2.5%) presented to the orthopaedic fracture clinic as a direct result of their abuse [11]. This study also reported that none of the women included in this study were asked about IPV by their attending orthopaedic surgeon. This is the first study that attempted to ascertain the number of women reporting to orthopaedic fracture clinics who were victims of IPV. The estimate is similar to previously reported one-year rates in other medical specialties, including internal medicine, pediatrics, and obstetrics and gynecology [20].

### Knowledge gaps among orthopaedic surgeons

Knowledge and comfort with IPV identification has been assessed in large scale surveys and smaller qualitative studies. Of 186 surgeon members of the Canadian Orthopaedic Association surveyed, 148 (80%) believed that IPV was exceedingly rare among the women they treated, affecting less than one percent of their patients [8]. Furthermore, one in two surgeons expressed that they lacked knowledge of the appropriate resources available to IPV victims (53%). Many surgeons held a number of misperceptions about IPV including 1) victims must be getting something out of the abusive relationships (14%); 2) some women have personalities that cause the abuse (20%); and 3) the battering would stop if the batterer quit abusing alcohol (43%) [8]. These findings were supported by a similar evaluation of US surgeon members of the Orthopaedic Trauma Association which found that among 153 surgeon members of the Orthopaedic Trauma Association, several misconceptions were evident: 1) victims must be getting something out of the abusive relationships (16%); 2) some women have personalities that cause the abuse (20%); and 3) the battering would stop if the batterer quit abusing alcohol (40%). In the past year, only 4% of respondents currently screened for IPV among female patients with injuries [9].

Whether the knowledge gaps resulted from a lack of reinforcing early education during medical school and residency training into later practice or instead, a systemic lack of education in the system is unclear. However, a recent online survey demonstrated knowledge gaps and discomfort in both surgical residents and medical students [10]. Respondents reported feelings that physicians should not interfere with a couple’s conflicts (21%), that patient’s personalities caused them to be abused (41%), and the majority (84% of medical students and 60% of surgical residents) felt that their training on IPV was inadequate [10]. Over 90% of both residents and medical students estimated the prevalence of IPV in their intended practice is less than 10% [10].

### How can we help IPV victims in the orthopaedic fracture clinic setting

The varied research exploring the relationship between IPV and orthopaedics allows for greater understanding of the issue at hand and helps to generate possible responses to this significant public health issue. The demonstrated high prevalence of IPV among patients presenting to orthopaedic fracture clinics [11], suggests that fracture clinics are an opportune setting for offering support to IPV victims. There is reason to believe that patients in orthopaedic clinics may experience more severe IPV than other types of patients because their fractures and other injuries warrant an orthopaedic surgeon’s care. Furthermore, IPV victims may not be effectively assisted by other health care professionals (HCPs), so asking about IPV at the level of the fracture clinic may be a window of opportunity. For example, a study of family medicine records showed that fewer than 15% of abused women had their abuse documented in a medical chart [21].

There are multiple initiatives that could be implemented within the orthopaedic fracture clinic setting to help IPV victims and provide them with the appropriate social support. Prior to the widespread implementation of any new initiative or program, it should be appropriately evaluated, following an evidence-based approach. Several of these programs are currently underway and are described in the next section. There are also simple steps that orthopaedic surgeons can take immediately and they are outlined in Table 1.

**Table 1:** Roles and responsibilities of orthopaedic surgeons (adapted from the canadian orthopaedic association position statement on IPV - version 2 – december 2012)

**Domain**	**Simple things surgeons can do**
*Education and Awareness*	• Educate yourself about IPV.
	• Consider IPV when diagnosing and treating patients.
	• Be aware that disclosure is a voluntary act, and, therefore, the decision to disclose or not disclose must be respected.
	• Be knowledgeable about counseling, shelters and social and legal services that are available locally and have hospital and community support contact information readily available, as well as toll-free help-lines for IPV. These resources are location-dependent but the USA and Ontario toll-free help line numbers are below:
	National Domestic Violence Hotline (USA): 1-800-799-SAFE
	Assaulted Women’s Helpline (Ontario): 1-866-863-0511
*Asking About IPV*	• Bring up IPV in a conversational manner: *“Because violence is so common in many people’s lives and because there is help available for people being abused, I now ask every patient about domestic violence. Is this something that is happening in your life?”*
	• Follow up with the three direct questions: “*Have you been physically abused by an intimate partner?”,* “*Have you been emotionally abused by an intimate partner?”,* “*Have you been sexually abused by an intimate partner?”* OR use a mobile phone app to assist in screening.
	• A statement — such as, *“I’d rather risk offending you than miss the opportunity to provide you with some information or possible resources that could help you in the future.”* — can be very helpful in initiating a referral to social services and moving beyond the purely medical context.
*After Disclosure*	• If the patient discloses IPV validate their feelings by telling them that the abuse is not their fault. Be non-judgmental, empathic and supportive throughout the interaction.
	• Assess the patient’s safety (and the safety of any children) in the home. *“Do you feel safe returning home today?”*
	• If the patient feels unsafe, and with her/his permission, initiate a safety strategy immediately through referral to social services or shelter as required.
	• Provide care for the patient’s immediate injuries and orthopaedic-related issues.
	• Take clear, legible, objective clinical notes, using the patient’s own words about abuse. Add diagrams or photographs, when appropriate. Should the patient be unwilling to talk about how the injuries were sustained or about the possibility of IPV, documentation and your impressions could be of benefit to the patient sometime in the future.
	• Provide a referral and contact information for local hospital-based or community-based support services if the patient is open to it.
	• In Canada, physicians are not legally obligated to report abuse of adults to the police. In some US states reporting of IPV is mandatory. Ensure that you know the legal requirements for your jurisdiction.
	• If you believe that children are at risk, you must notify your local Child Protective Services agency. Ensure you know the reporting requirements for your jurisdiction.

### Practical approaches to identification and support of IPV victims in a clinic setting

**Figure 1:**
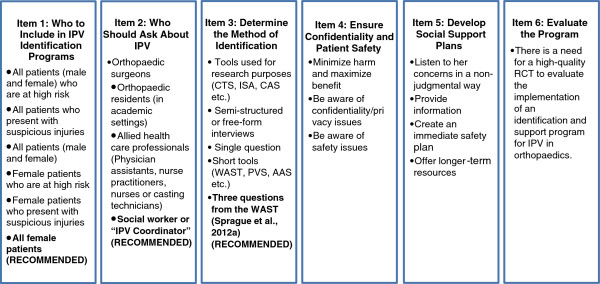
Developing and evaluating an IPV identification and support program – the initial steps.

Multiple system-level factors must be considered to enable orthopaedic surgeons to effectively identify and help patients who have experienced IPV, including case-finding protocols and support plans. Multiple items need to be carefully considered when developing an IPV case-finding protocol in the fracture clinic setting, which are described below and summarized in Figure 1. An evidence-based approach should be followed when possible.

Item 1: Determine Who Should be Asked - A Case for Case-Finding in Trauma

Multiple methods of IPV identification have been developed and evaluated. In universal screening, HCPs routinely ask all patients who presented to them about abuse, regardless of whether they show signs of IPV or are considered at risk of IPV [22]. In a case-finding approach, HCPs ask only patients that they believe may be victims of IPV about abuse or those who are deemed to be high risk [22]. One of the limitations of case-finding is that HCPs may not be able to accurately determine who the victims of IPV are and they may miss multiple opportunities to help victims [22]. We argue that asking about IPV in orthopaedic trauma is targeted “case-finding” as opposed to “universal screening” because evidence demonstrates that orthopaedic trauma patients experience a similar prevalence of IPV compared to other medical specialties [13] but it could be more severe because IPV that is seen in orthopaedics has often escalated to the point of causing major orthopaedic injuries such as fractures and dislocations [13]. This means that we would be asking a population that has a demonstrated high risk of IPV, in contrast to asking all women who present to emergency departments, for example, since emergency department patients have a large range of health care issues that may or may not be indicative that they are at high risk for IPV.

While who to include in an IPV identification program within an orthopaedic fracture clinic setting may seem like a simple question, there are multiple options and the best approach has not been established. Primary options are shown in Figure 1.

Although, on the surface, it may seem fair to ask all men about IPV in addition to all women, evidence shows that women are disproportionately affected by IPV compared to men [23-25]. Men are also less likely to admit that they are victims of abuse and less likely to seek help, making identifying IPV in men a greater challenge [26]. Since most research to date, and all IPV research in surgery, focuses on violence against women, more research is required to determine the best approach to identifying male victims of IPV as well as determining if asking men about IPV is effective, practical, and cost-efficient. We argue that it is unethical to implement an IPV screening program for men without having appropriate supports in place to support men who have experienced IPV. In many jurisdictions, men are underserved when it comes to IPV supports [26]. Future studies should investigate the best ways to ask about IPV against men as well as implementing support programs so Health Care Providers (HCPs) are able to refer men for assistance when required.

It is difficult to define who is at high risk and researchers have not yet been able to construct a sensitive model of how an IPV victim presents for medical care [27-29]. HCPs tend to screen for IPV based on stereotypes of patients who they think are at high risk [30]. Since IPV is present across all ethnicities, socioeconomic statuses, and relationship types [15], these stereotypes are often incorrect or incomplete. It is plausible that HCPs may overlook a high proportion of cases if they screen solely based on their perceptions and stereotypes. If asking about IPV were a part of a routine medical assessment in the fracture clinic, patients may become accustomed to being asked about IPV and it would be seen as a normal discussion, much like being asked about sexual history or smoking status [22]. Women may feel more comfortable speaking to their HCP about IPV, facilitating disclosure. Based on the above, our initiative would include all women who present to the fracture clinic in an IPV case-finding program.

Item 2: Determine Who Should Ask about IPV

The majority of women support asking about IPV in a health care setting and 95% of patients would prefer to disclose to an HCP as opposed to a friend, family member, or coach [30]. Multiple HCPs are well positioned to ask about IPV and there are advantages and disadvantages associated with each option. In the orthopaedic fracture clinic setting, the individuals who could ask about IPV are listed in Figure 1.

Orthopaedic surgeons interact with up to 100 patients a day in a busy fracture clinic. A major barrier to asking about IPV, according to HCPs, is that they often have little to no training on how to talk to patients about IPV or how to refer victims to other resources [31].

Allied HCPs such as physician assistants, nurse practitioners, nurses, or casting technicians could be another option. Although Gerlach et al. report that gender of the IPV screener is not associated with disclosure rates [32], many women report that they would feel more comfortable being asked about IPV by a female HCP [30]. Nurses and casting technicians are often female, which is a potential advantage; however they are just as busy as surgeons and residents with their existing responsibilities already. A lack of training may be an additional issue [33]. It would take a great deal of institutional restructuring to allow any of these busy allied HCPs to dedicate the required time for the IPV screening process.

Another option would to have designated HCPs a specially trained nurse, social worker, or other highly trained individual be available to identify and offer assistance to victims of IPV. This option could potentially eliminate many of the barriers that HCPs currently face. One problem with having an outside person involved is that it is resource-intensive. However, there is already an example of a successful campaign to have an outside person in orthopaedic fracture clinics that is analogous. Osteoporosis used to be a perceived as a purely medical problem that orthopaedic surgeons rarely treated directly. Now, many orthopaedic centres in Canada have an osteoporosis coordinator in orthopaedic fracture clinics to screen and treat patients with osteoporosis, and this has proven to be a sustainable program [34]. While this is a medical model, and IPV is a social issue, we believe that similar to this osteoporosis model, having a specially-trained “IPV Coordinator” available in orthopaedic clinics to identify victims of IPV and offer appropriate assistance may be an ideal approach.

Item 3: Determine the Method of Identification

Multiple tools have been developed to identify victims of IPV for both research and clinical uses and the levels of validation vary across the different instruments.

Instruments used for research purposes, such as the Index of Spouse Abuse (ISA) [35] and Conflict Tactics Scale (CTS) [36] and Composite Abuse Scale (CAS) [37], are often lengthy questionnaires. Although these tools are well validated and highly used as gold standards in the research setting to validate other scales [38], they would be very difficult to administer in a busy clinic environment due to logistics of time and scoring. The semi-structured or free-form interview approach may not work in orthopaedic clinics because it requires a lot of training and may be too time consuming to administer.

Screening tools with a single question are inconsistent in their ability to detect victims according to a review by Rabin et al. so they should not be used [38]. For example, Peralta et al. used the question “In the past 3 months, did you feel safe at home?” to evaluate the prevalence of IPV and found that the sensitivity was only 8.8% and the specificity was 91.2% [39]. Similarly, Sagrestano et al. used the question “Are you suffering mental or physical abuse now?” to assess the prevalence of IPV [40]. They found that only 3% of women answered affirmatively to the single question as opposed to 17% of women on the longer Conflict Tactics Scale.

**Figure 2:**
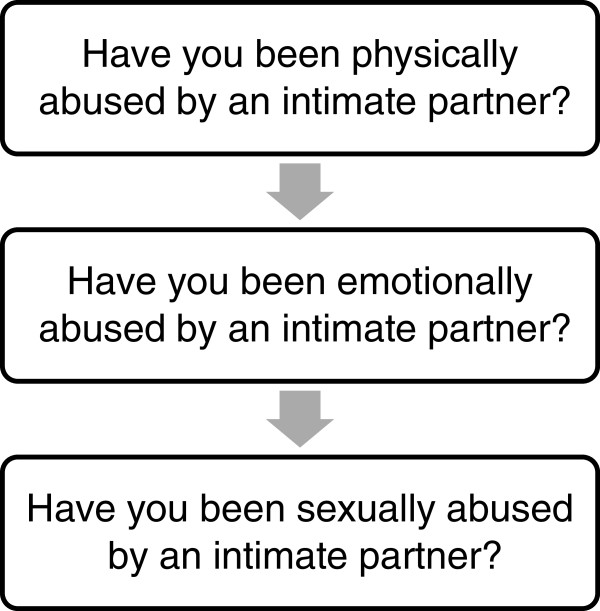
Recommended IPV screening questions for orthopaedics (a subset of the WAST).

Several shorter tools that were designed to be used in clinic are widely used. The Woman Abuse Screening tool (WAST) [41], Partner Violence Screen (PVS) [42], and Abuse Assessment Screen (AAS) [43] are among the most widely used and validated short screening tools [38]. In orthopaedic trauma populations, previous research has found that the WAST and PVS have very good specificity, but the sensitivity is relatively low [12]. It is important that we maximize sensitivity with IPV screening tools to avoid missing the opportunity to assist victims. Although good specificity is fairly important, having a good set of questions to begin with is essential from which HCPs can initiate a more in-depth conversation with the patient [12]. Although these tools are frequently used in other settings, there is a subset of the WAST questions that have increased sensitivity in the orthopaedic trauma population [12] (Figure 2). A simple set of three questions is easy to remember, does not take a lot of time, and the results can be determined easily, not with a complicated scoring procedure. In addition, the American Medical Association and the Canadian Orthopaedic Association recommend using direct questions because they are easy to understand and tend to elicit direct responses [15,44].

Another simple measure that surgeons and other HCPs should consider is the mobile phone app called “R3” made by Harbour House shelter in Florida that guides HCPs step-by-step through the process of asking about IPV in a medical setting using the well-studied HITS screening tool [45]. The app also provides some local, state, and national (USA) referral resources for victims of IPV and it also provides some specific instructions such as ensuring the victim is connected with an advocate before leaving the appointment (if available), using the patient’s own words to document abuse, and tips on how to assess safety and follow up appropriately. Future endeavours could include expanding upon available apps to include resources for other countries or developing new apps for specific purposes such as screening in orthopaedics specifically.

Item 4: Ensure Confidentiality and Patient Safety in the Clinic Setting

When discussing IPV, it is critical that HCPs minimize the victims’ risk of harm and maximize potential benefits [17]. Discussing IPV could have negative consequences for IPV victims including anxiety, shame, fear, and physical harm [46]. These risks can be minimized through careful consideration of the type of environment in which screening takes place. Maintaining the confidentiality of IPV victims is of paramount importance because of the sensitive nature of the topic [47]. The subject should not be broached with the patient’s partner, friends, or family in the area, to respect the patient's safety and confidentiality [17]. The vast majority of patients agree that it is important to ask about IPV in a private location [30]. Creating an environment conducive to maintaining confidentiality may be challenging; one of the most commonly reported barriers to screening for IPV is that clinics are not private enough [31]. Many fracture clinics (especially in older hospitals) are not constructed with having a personal and delicate conversation in mind. However, in even the most open clinics, there is often a room or small space with a bit more privacy which an IPV coordinator could use to speak to patients about IPV, thereby reducing the likelihood of harm. Victims should be treated in a manner that will minimize their anxiety, shame, and fear, assuring them that their abuse is not their fault.

Item 5: Develop Social Support Plans

When assisting with a complex issue such as IPV, the victim’s choices must be respected [17]. If a patient does not wish to disclose about an IPV experience, the HCP should understand that it is the patient’s choice and not to force the conversation. Similarly, if the patient chooses to disclose but not take action (leave the relationship, accept referrals, seek counselling etc.), then the patient’s wishes should be respected. IPV is a very complex issue that involves social, financial, physical, and psychological considerations and consequences. Following disclosure, it may be difficult for an IPV victim to accept help for fear of losing their children, losing their partner, being financially worse off, or experiencing retaliation from their partner [48]. IPV management must be individualized, effectively addressing each victim’s particular needs [17]. The IPV Coordinator must be sufficiently experienced with assisting victims of IPV and must be aware of the complex social, psychological, financial, and familial issues that surround IPV to help to provide IPV victims with the appropriate social support.Health care guidelines for implementing IPV identification and support programs include the following components to assist women who have experienced IPV: 1) Listen to her concerns in a non-judgmental way; use phrases like “I am concerned for your safety” or “The abuse is not your fault”, 2) Provide information that helps to reduce misconceptions and alleviate fear and anxiety, 3) Create an immediate safety plan with the patient; make sure that she is safe to go home and offer immediate assistance such as a social worker or legal/police assistance if required and if she agrees, 4) Offer longer-term resources such as health-care or community support system access, women’s shelters, other specialized local services, etc. [17].

Item 6: Evaluate the Program

The next step in advancing IPV advocacy in orthopaedic fracture clinics is to pilot and evaluate a universal screening program within the orthopaedic fracture clinic setting. This program would include having a trained IPV Coordinator to ask three screening questions (Figure 2) to all patients who present to the orthopaedic fracture clinic. With patients who screen positive, the IPV coordinator would assist the patient with providing the appropriate social support as described above. This is a challenging and costly initiative to both implement and evaluate. The cluster randomized controlled trial study design may be the most appropriate because the intervention would be meant to be applied at the clinic level as opposed to the individual level. Cluster randomized designs are best for when the unit of randomization is a clinic, school, family, or other easily defined group [49].

### Future directions - bridging the gaps in knowledge and research

The current literature has identified numerous gaps in both IPV knowledge and research in the field of orthopaedic surgery. Multiple research initiatives are currently underway and are being developed to further advance this important field which include assessing the prevalence of IPV, evaluating an IPV fracture clinic tool kit, and developing and evaluating an IPV case-finding program in the orthopaedic setting. Each of these is described in detail below.

### Assessing the prevalence of IPV

A larger multi-centre IPV prevalence study is currently being conducted at eleven sites in North America, Europe, and Asia [13]. Approximately 3000 women will be included in this initiative. This study will provide a more accurate estimate of the prevalence of IPV in the orthopaedic fracture clinic setting and will demonstrate the differences in prevalence rates between nations [13]. The results of this study will further inform the need for IPV screening and offering victim’s support programs within the orthopaedic fracture clinic setting.

### Providing an IPV fracture clinic tool kit

Another way that fracture clinic personnel may be able to help IPV victims is through an IPV “toolkit” that includes IPV awareness posters, buttons, and pamphlets to post throughout the fracture clinic setting. The Family Violence Prevention Project found that the implementation of pamphlets, resource cards and examination room posters increased the number of clinician referrals and patient self-referrals to an on-site domestic violence evaluator more than twofold [50], indicating that such an intervention may have a significant impact on a patient’s willingness to discuss IPV in the clinic. HCPs can also be provided with a simple set of steps to follow in case of disclosure. Previous research has shown that orthopaedic surgeons, medical students, and surgical residents are largely unsure of what to do if a patient discloses [8-10]. A pre- and post-interventional study is currently under way that aims to evaluate whether the presence of posters, buttons, and pamphlets on IPV changes patients perceptions about IPV and their comfort level with discussing IPV within the orthopedic setting.

### Developing and evaluating an IPV identification program – the initial steps

Identifying victims of IPV within the fracture clinic setting through a case-finding program may be another method of assisting individuals who are being abused. Multiple researchers have suggested that screening for IPV in a medical setting is not supported by evidence [51,52] and “universal screening” for IPV within medical settings remains highly controversial, as is exemplified in the debate between Wathen and MacMillan, and Taket [22]. MacMillan and Wathen hold the opinion that routinely screening all women for IPV in any setting (universal screening) is not appropriate and has potential harms [22]. They support targeted “case-finding” for women who present with certain signs and symptoms of IPV [22]. Conversely, Taket holds the opinion that universal screening should be applied because it “contributes to changing social attitudes to domestic abuse” among other benefits such as decreasing stigmatization, possible increased safety compared to selective screening, and avoiding incorrect stereotypes of IPV among HCPs [22].

A recent randomized controlled trial on identification of IPV in primary care clinics concluded that screening all women for IPV did not increase quality of life [53]. This trial had three intervention groups: a group that was screened using a computer and received a list of local resources, a group that received a list of local resources only, and a group that received no intervention. This study, along with MacMillan and colleagues’ randomized trial shows that passive interventions are not effective [51]. This evidence suggests there is a need for a high quality trial evaluating “active” screening or case finding programs that include both an identification component and a component where women who disclose can get the support they require.

## Conclusions

Based on the available evidence in surgery and other medical fields, surgeons should recognize that IPV is a serious public health issue that affects a large proportion of orthopaedic patients. Surgeons and other HCPs should be aware of the various issues and complexities surrounding the problem of IPV. We propose a stepwise, structured approach to developing a support program that includes the following: Item 1) Decide who to include in IPV identification programs; Item 2) Determine who should ask about IPV; Item 3) Determine the method of identification; Item 4) Ensure confidentiality and patient safety in the clinic setting; Item 5) Develop social support programs; Item 6) Evaluate the program. Additional research is currently underway to inform the development of an IPV screening program within the orthopaedic fracture clinic setting.

## Competing interests

The authors declare that they have no competing interests.

## Authors’ contributions

All authors contributed to the study design and manuscript preparation. All authors read and approved the final manuscript.

## Pre-publication history

The pre-publication history for this paper can be accessed here:

http://www.biomedcentral.com/1471-2474/14/23/prepub
